# Crystal structure and Hirshfeld surface analysis of 3-[(1*E*)-(4-{4-[(*E*)-(3-hy­droxy­benzyl­idene)amino]­phen­oxy}phenyl­imino)­meth­yl]phenol

**DOI:** 10.1107/S205698902100181X

**Published:** 2021-02-19

**Authors:** Shaaban K. Mohamed, Joel T. Mague, Mehmet Akkurt, Farouq E. Hawaiz, Sahar M. I. Elgarhy, Elham A. Al-Taifi

**Affiliations:** a Chemistry and Environmental Division, Manchester Metropolitan University, Manchester, M1 5GD, England; b Chemistry Department, Faculty of Science, Minia University, 61519 El-Minia, Egypt; c Department of Chemistry, Tulane University, New Orleans, LA 70118, USA; d Department of Physics, Faculty of Sciences, Erciyes University, 38039 Kayseri, Turkey; e Chemistry Department, College of Education, Salahaddin University-Hawler, Erbil, Kurdistan Region, Iraq; f Faculty of Science, Department of Biochemistry, Beni Suef University, Beni Suef, Egypt; g Department of Chemistry, Faculty of Science, Sana’a University, Sana’a, Yemen

**Keywords:** crystal structure, hydrogen bond, phenol, aromatic ether, phen­oxy, azomethines, Hirshfeld surface analysis

## Abstract

In the crystal, the mol­ecule of the title compound, C_26_H_20_N_2_O_3_, has crystallographically imposed twofold rotation symmetry. The crystal packing consists of layers parallel to the *ab* plane formed by O—H⋯N and C—H⋯O hydrogen bonds. Between the layers, C—H⋯π inter­actions are observed.

## Abstract 2

In the crystal, the mol­ecule of the title compound has crystallographically imposed twofold rotation symmetry. The crystal packing consists of layers parallel to the *ab* plane formed by O—H⋯N and C—H⋯O hydrogen bonds.

## Chemical context   

Several Schiff bases have been reported for their significant biological activities such as anti­tumor (Mansouri *et al.*, 2013 ▸), anti-inflammatory (Shukla & Mishra, 2019 ▸), anti­bacterial (Van Zee & Coates, 2015 ▸) or anti­microbial (Pagadala *et al.*, 2015 ▸). Schiff bases are also used as versatile components in nucleophilic addition with organometallic reagents and in cyclo­addition reactions (Mohan *et al.*, 2012 ▸). These findings prompted us to investigate the crystal structure of the title compound.
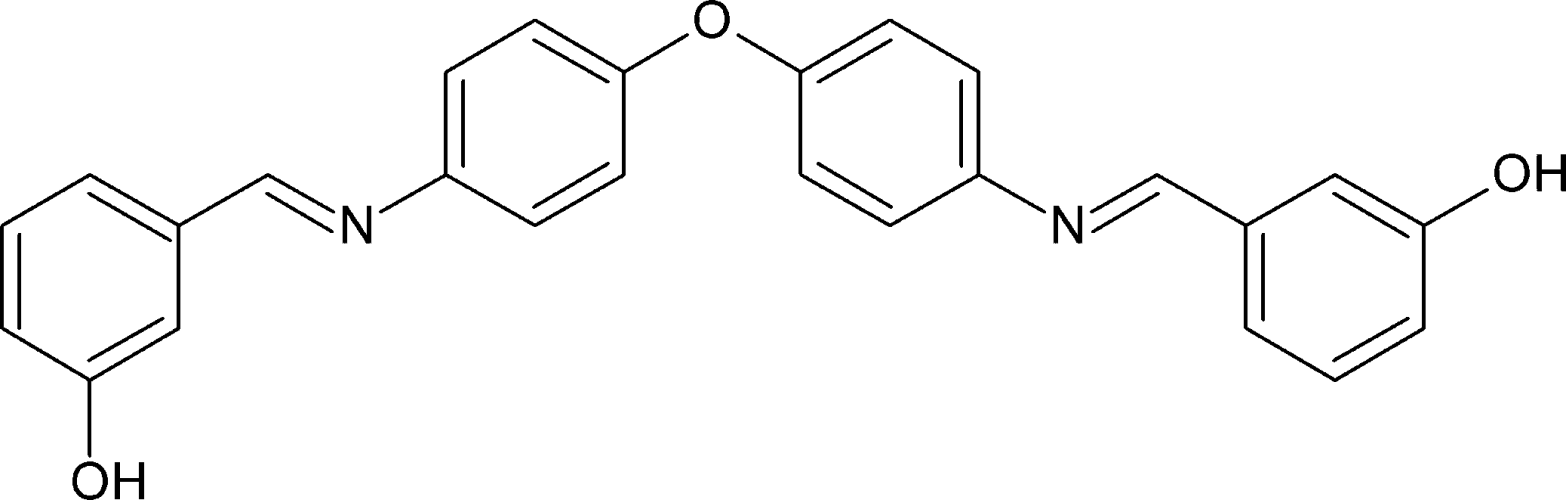


## Structural commentary   

**Figure 1:**
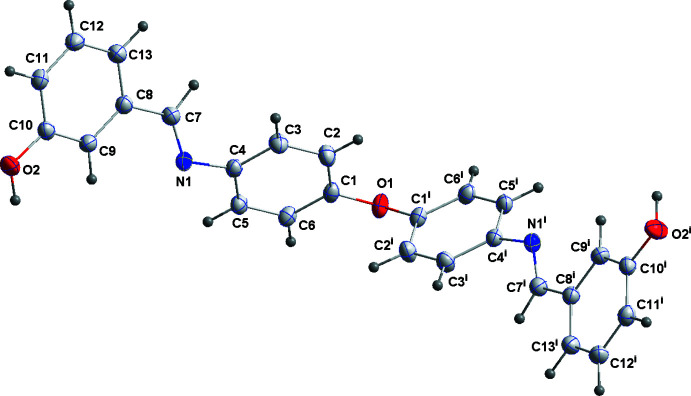
The title mol­ecule with labeling scheme and 50% probability ellipsoids [symmetry code: (i) −*x* + 1, *y*, −*z* + 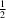].

The mol­ecule of the title compound has crystallographically imposed twofold rotation symmetry (Fig. 1 ▸). The dihedral angle between the two unique benzene rings is 40.68 (6)° while the dihedral angle between the two central benzene rings is 77.71 (6)°. Bond lengths are typical for this kind of compounds.

## Supra­molecular features   

**Figure 2:**
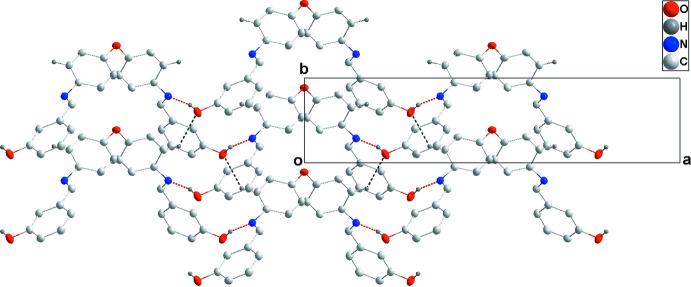
The layer structure viewed along the *c*-axis direction. The inter­molecular O—H⋯N and C—H⋯O hydrogen bonds are shown as red and black dashed lines, respectively.

**Figure 3:**
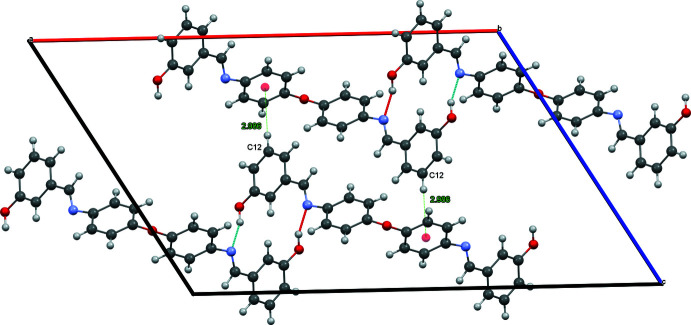
Side view of two layers seen along the *b*-axis direction. Hydrogen bonds and C—H⋯π inter­actions are depicted by dashed lines.

In the crystal, O2—H2*A*⋯N1 and C5—H5⋯O2 hydrogen bonds link the mol­ecules into layers parallel to the *ab* plane (Table 1 ▸, Fig. 2 ▸). The layers are hold together by C—H⋯π contacts (Table 1 ▸, Fig. 3 ▸) and by other van der Waals inter­actions (Table 2 ▸).

## Hirshfeld surface analysis   

**Figure 4:**
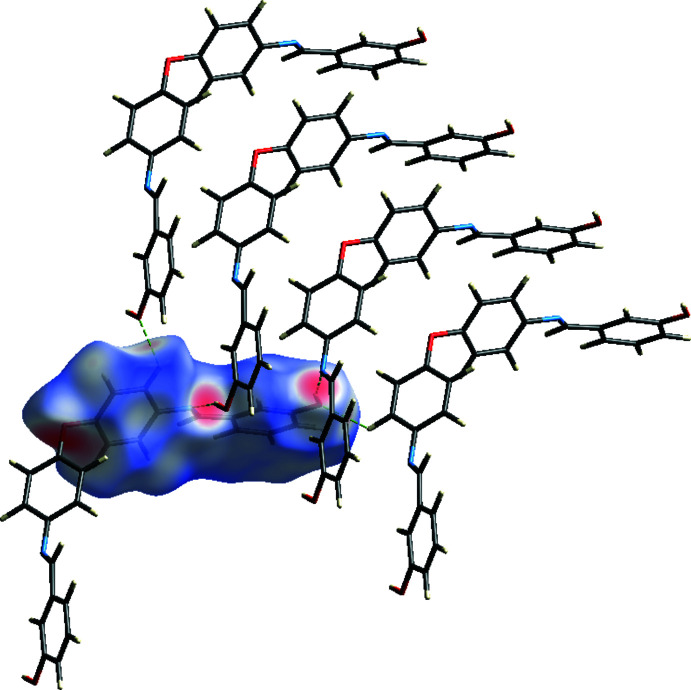
A view of the three-dimensional Hirshfeld surface for the title compound, plotted over *d*_norm_ in the range −1.1242 to 1.4437 a.u.

Hirshfeld surface analysis, together with two-dimensional fingerprint plots, is an important tool for visualizing and analyzing inter­molecular contacts in mol­ecular crystals. The corresponding surfaces and fingerprint plots were prepared by *CrystalExplorer* (Turner *et al.*, 2017 ▸). Fig. 4 ▸ shows the *d*_norm_ map for the title mol­ecule, with red spots indicating the positions of H⋯N contacts arising from the O—H⋯N hydrogen bonds.

**Figure 5:**
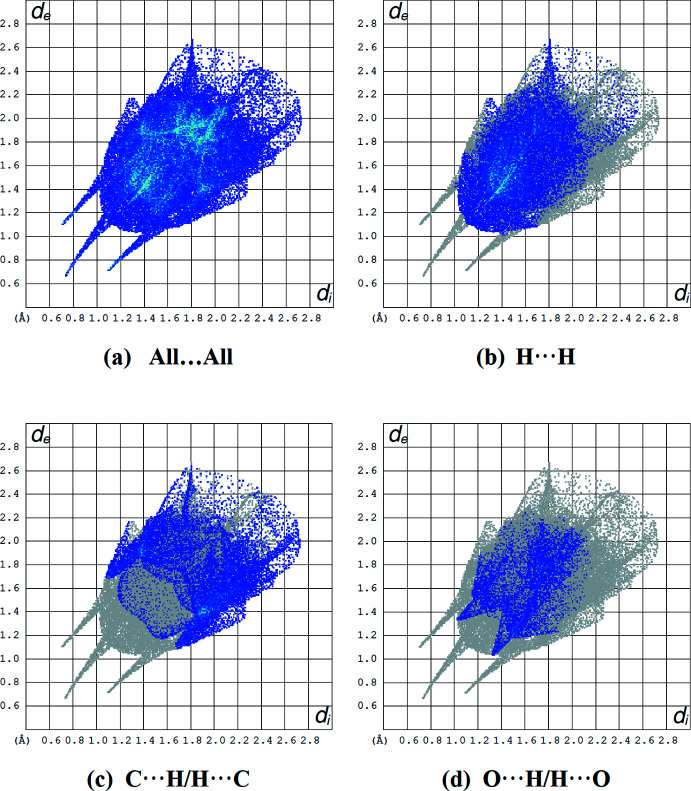
A view of the two-dimensional fingerprint plots for the title compound, showing (*a*) all inter­actions, and delineated into (*b*) H⋯H, (*c*) C⋯H/H⋯C and (*d*) O⋯H/H⋯O inter­actions. The *d*_i_ and *d*_e_ values are the closest inter­nal and external distances (in Å) from given points on the Hirshfeld surface.

Fig. 5 ▸ shows the two-dimensional fingerprint plots, which give the contributions of inter­molecular contacts to the Hirshfeld surface. The most important contribution to the Hirshfeld surface (41.6%) is from H⋯H contacts. C⋯H/H⋯C and O⋯H/H⋯O inter­actions follow with 28.1% and 13.8% contributions, respectively. Other minor contributors are C⋯C (5.3%), N⋯H/H⋯N (4.8%), O⋯C/C⋯O (3.8%) and N⋯C/C⋯N (2.6%) contacts.

## Database survey   

Five related compounds with a 4-[(*E*)-benzyl­idene­amino]­phenol skeleton are: (*E*)-2-{[(2-amino­phen­yl)imino]­meth­yl}-5-(benz­yloxy)phenol (NIBRIC; Ghichi *et al.*, 2018 ▸), (*Z*)-3-(benz­yloxy)-6-{[(5-chloro-2-hy­droxy­phen­yl)amino]­methyl­idene}cyclo­hexa-2,4-dien-1-one (NIBROI; Ghichi *et al.*, 2018 ▸), 2-{(*E*)-[(2-methyl-3-nitro­phen­yl)imino]­meth­yl}-4-nitro­phenol (AFOPUI; Tanak *et al.*, 2013 ▸), 2-[(*E*)-(2-chloro­phen­yl)imino­meth­yl]-6-methyl­phenol (SABKOX; Zhu *et al.*, 2010 ▸) and 2-{[(2,4-di­methyl­phen­yl)imino]­meth­yl}-6-methyl­phenol (MUCDIY; Tanak *et al.*, 2009 ▸).

In the crystal of NIBRIC, strong N—H⋯O hydrogen bonds form zigzag chains of mol­ecules along the *b*-axis direction. Weaker C—H⋯π and offset π–π stacking inter­actions also contribute to the packing. For NIBROI, pairs of strong O—H⋯O hydrogen bonds form centrosymmetric dimers that enclose 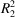
(18) rings. These combine with weaker C—H⋯Cl hydrogen bonds, which also generate centrosymmetric dimers, but with 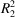
(14) motifs. Inversion-related C—H⋯π contacts lead to the formation of sheets of mol­ecules parallel to (120), which are stacked approximately along the *b*-axis direction. In the crystal of AFOPUI, mol­ecules are linked by C—H⋯O inter­actions, forming two-dimensional sheets parallel to the *bc* plane. In the structure of SABKOX, the hy­droxy H atom is involved in a strong intra­molecular O—H⋯N hydrogen bond, generating a *S*(6) ring. The mol­ecular structure of MUCDIY is stabilized by an intra­molecular O—H⋯N hydrogen bond, which generates a six membered ring.

## Synthesis and crystallization   

Condensation of 1 mmol of 4,4′-oxydibenzaldehyde (226 mg) with 2 mmol of 3-amino­phenol (218 mg) in ethanol under reflux for 4 h afforded the crude product of the title compound. The product was crystallized from ethanol by slow evaporation to obtain good quality crystals for X-ray diffraction. Yield 82%.

## Refinement   

Crystal data, data collection and structure refinement details are summarized in Table 3 ▸. All H atoms were located in a difference-Fourier map and refined freely.

## Supplementary Material

Crystal structure: contains datablock(s) global, I. DOI: 10.1107/S205698902100181X/yk2146sup1.cif

Structure factors: contains datablock(s) I. DOI: 10.1107/S205698902100181X/yk2146Isup2.hkl

Supporting information file. DOI: 10.1107/S205698902100181X/yk2146Isup3.cml

CCDC reference: 2062957

Additional supporting information:  crystallographic information; 3D view; checkCIF report

## Figures and Tables

**Table 1:** Hydrogen-bond geometry (Å, °) *Cg*1 is the centroid of the C1–C6 benzene ring.

*D*—H⋯*A*	*D*—H	H⋯*A*	*D*⋯*A*	*D*—H⋯*A*
O2—H2*A*⋯N1^i^	0.972 (19)	1.828 (19)	2.7615 (12)	160.1 (16)
C5—H5⋯O2^ii^	0.973 (13)	2.431 (14)	3.1121 (14)	126.7 (10)
C12—H11⋯*Cg*1^ii^	1.004 (14)	2.986 (15)	3.9882 (12)	178.7 (19)

Symmetry codes: (i) -x+{\script{1\over 2}}, y-{\script{1\over 2}}, -z+{\script{1\over 2}}; (ii) -x+{\script{1\over 2}}, y+{\script{3\over 2}}, -z+{\script{1\over 2}}.

**Table 2:** Short inter­molecular contacts (Å) in the title structure

Contact	Distance	Symmetry operation
H12⋯O1	2.763 (14)	1 − *x*, 2 − *y*, 1 − *z*
H6⋯H11	2.53 (2)	*x*, 2 − *y*, −{1\over 2} + *z*
C3⋯C6	3.5155 (15)	*x*, −1 + *y*, *z*
C6⋯H11	2.892 (15)	*x*, 1 − *y*, −{1\over 2} + *z*
C11⋯C11	3.319 (2)	{1\over 2} − *x*, {1\over 2} − *y*, 1 − *z*
H11⋯H2	2.40 (3)	1 − *x*, 1 − *y*, 1 − *z*

**Table 3:** Experimental details

Crystal data	
Chemical formula	C_26_H_20_N_2_O_3_
*M*_r_	408.44
Crystal system, space group	Monoclinic, *C*2/*c*
Temperature (K)	150
*a*, *b*, *c* (Å)	26.8396 (6), 5.1174 (1), 17.2574 (4)
β (°)	121.764 (1)
*V* (Å^3^)	2015.27 (8)
*Z*	4
Radiation type	Cu *K*α
μ (mm^−1^)	0.72
Crystal size (mm)	0.25 × 0.06 × 0.06

Data collection	
Diffractometer	Bruker D8 VENTURE PHOTON 100 CMOS
Absorption correction	Multi-scan (*SADABS*; Krause *et al.*, 2015 ▸)
*T*_min_, *T*_max_	0.90, 0.96
No. of measured, independent and observed [*I* > 2σ(*I*)] reflections	15449, 1880, 1767
*R*_int_	0.031
(sin θ/λ)_max_ (Å^−1^)	0.609

Refinement	
*R*[*F* ^2^ > 2σ(*F* ^2^)], *wR*(*F* ^2^), *S*	0.033, 0.086, 1.05
No. of reflections	1880
No. of parameters	182
H-atom treatment	All H-atom parameters refined
Δρ_max_, Δρ_min_ (e Å^−3^)	0.19, −0.16

Computer programs: *APEX3* and *SAINT* (Bruker, 2016 ▸), *SHELXT* (Sheldrick, 2015*a* ▸), *SHELXL2016/6* (Sheldrick, 2015*b* ▸), *DIAMOND* (Brandenburg & Putz, 2012 ▸) and *SHELXTL* (Sheldrick, 2008 ▸).

## supplementary crystallographic information

### Crystal data

**Table:**

C_26_H_20_N_2_O_3_	*F*(000) = 856
*M**_r_* = 408.44	*D*_x_ = 1.346 Mg m^−^^3^
Monoclinic, *C*2/*c*	Cu *K*α radiation, λ = 1.54178 Å
*a* = 26.8396 (6) Å	Cell parameters from 9934 reflections
*b* = 5.1174 (1) Å	θ = 3.9–69.8°
*c* = 17.2574 (4) Å	µ = 0.72 mm^−^^1^
β = 121.764 (1)°	*T* = 150 K
*V* = 2015.27 (8) Å^3^	Column, colourless
*Z* = 4	0.25 × 0.06 × 0.06 mm

### Data collection

**Table:**

Bruker D8 VENTURE PHOTON 100 CMOS diffractometer	1880 independent reflections
Radiation source: INCOATEC IµS micro–focus source	1767 reflections with *I* > 2σ(*I*)
Mirror monochromator	*R*_int_ = 0.031
Detector resolution: 10.4167 pixels mm^-1^	θ_max_ = 69.8°, θ_min_ = 3.9°
ω scans	*h* = −32→32
Absorption correction: multi-scan (*SADABS*; Krause *et al.*, 2015)	*k* = −6→6
*T*_min_ = 0.90, *T*_max_ = 0.96	*l* = −20→20
15449 measured reflections	

### Refinement

**Table:**

Refinement on *F*^2^	Secondary atom site location: difference Fourier map
Least-squares matrix: full	Hydrogen site location: difference Fourier map
*R*[*F*^2^ > 2σ(*F*^2^)] = 0.033	All H-atom parameters refined
*wR*(*F*^2^) = 0.086	*w* = 1/[σ^2^(*F*_o_^2^) + (0.043*P*)^2^ + 1.3008*P*] where *P* = (*F*_o_^2^ + 2*F*_c_^2^)/3
*S* = 1.05	(Δ/σ)_max_ < 0.001
1880 reflections	Δρ_max_ = 0.19 e Å^−^^3^
182 parameters	Δρ_min_ = −0.16 e Å^−^^3^
0 restraints	Extinction correction: *SHELXL* 2016/6 (Sheldrick, 2015*b*), Fc^*^=kFc[1+0.001xFc^2^λ^3^/sin(2θ)]^-1/4^
Primary atom site location: structure-invariant direct methods	Extinction coefficient: 0.0031 (2)

### Special details

**Table:**

Geometry. All esds (except the esd in the dihedral angle between two l.s. planes) are estimated using the full covariance matrix. The cell esds are taken into account individually in the estimation of esds in distances, angles and torsion angles; correlations between esds in cell parameters are only used when they are defined by crystal symmetry. An approximate (isotropic) treatment of cell esds is used for estimating esds involving l.s. planes.
Refinement. Refinement of F^2^ against ALL reflections. The weighted R-factor wR and goodness of fit S are based on F^2^, conventional R-factors R are based on F, with F set to zero for negative F^2^. The threshold expression of F^2^ > 2sigma(F^2^) is used only for calculating R-factors(gt) etc. and is not relevant to the choice of reflections for refinement. R-factors based on F^2^ are statistically about twice as large as those based on F, and R- factors based on ALL data will be even larger.

### Fractional atomic coordinates and isotropic or equivalent isotropic displacement parameters (Å^2^)

**Table:**

	*x*	*y*	*z*	*U*_iso_*/*U*_eq_	
O1	0.500000	1.3874 (2)	0.250000	0.0267 (3)	
O2	0.21618 (3)	0.09976 (18)	0.33558 (6)	0.0336 (2)	
H2A	0.1966 (8)	0.182 (4)	0.2759 (13)	0.066 (5)*	
N1	0.36195 (4)	0.78690 (18)	0.33726 (6)	0.0233 (2)	
C1	0.46708 (5)	1.2402 (2)	0.27573 (7)	0.0228 (3)	
C2	0.49250 (5)	1.0380 (2)	0.33777 (8)	0.0288 (3)	
H2	0.5331 (6)	0.994 (3)	0.3625 (9)	0.035 (3)*	
C3	0.45903 (5)	0.8917 (2)	0.36148 (8)	0.0276 (3)	
H3	0.4769 (6)	0.743 (3)	0.4038 (9)	0.033 (3)*	
C4	0.39966 (4)	0.9482 (2)	0.32294 (7)	0.0220 (3)	
C5	0.37512 (5)	1.1545 (2)	0.26182 (7)	0.0244 (3)	
H5	0.3337 (6)	1.192 (3)	0.2353 (9)	0.031 (3)*	
C6	0.40866 (5)	1.3034 (2)	0.23883 (7)	0.0242 (3)	
H6	0.3914 (5)	1.445 (3)	0.1965 (9)	0.030 (3)*	
C7	0.38127 (5)	0.6916 (2)	0.41641 (7)	0.0254 (3)	
H7	0.4211 (6)	0.744 (3)	0.4696 (10)	0.035 (4)*	
C8	0.35036 (5)	0.4960 (2)	0.43754 (7)	0.0238 (3)	
C9	0.29481 (5)	0.4001 (2)	0.37197 (7)	0.0242 (3)	
H9	0.2735 (5)	0.460 (3)	0.3094 (9)	0.026 (3)*	
C10	0.26950 (5)	0.2038 (2)	0.39519 (7)	0.0242 (3)	
H10	0.2803 (6)	−0.038 (3)	0.4990 (9)	0.030 (3)*	
C11	0.29889 (5)	0.1028 (2)	0.48379 (8)	0.0262 (3)	
H11	0.3729 (6)	0.127 (3)	0.6118 (10)	0.034 (3)*	
C12	0.35320 (5)	0.2004 (2)	0.54851 (8)	0.0278 (3)	
H12	0.4190 (6)	0.465 (3)	0.5730 (9)	0.030 (3)*	
C13	0.37916 (5)	0.3963 (2)	0.52590 (8)	0.0268 (3)	

### Atomic displacement parameters (Å^2^)

**Table:**

	*U*^11^	*U*^22^	*U*^33^	*U*^12^	*U*^13^	*U*^23^
O1	0.0298 (6)	0.0206 (5)	0.0400 (6)	0.000	0.0254 (5)	0.000
O2	0.0266 (4)	0.0395 (5)	0.0285 (5)	−0.0111 (4)	0.0103 (4)	−0.0010 (4)
N1	0.0218 (5)	0.0235 (5)	0.0265 (5)	0.0009 (4)	0.0141 (4)	0.0018 (4)
C1	0.0256 (5)	0.0208 (5)	0.0278 (6)	−0.0023 (4)	0.0181 (5)	−0.0032 (4)
C2	0.0204 (5)	0.0313 (6)	0.0344 (6)	0.0025 (5)	0.0142 (5)	0.0046 (5)
C3	0.0240 (6)	0.0273 (6)	0.0304 (6)	0.0030 (5)	0.0136 (5)	0.0066 (5)
C4	0.0224 (5)	0.0226 (5)	0.0235 (5)	−0.0011 (4)	0.0138 (4)	−0.0019 (4)
C5	0.0221 (5)	0.0262 (6)	0.0275 (6)	0.0037 (4)	0.0148 (5)	0.0013 (4)
C6	0.0276 (6)	0.0215 (5)	0.0277 (6)	0.0042 (4)	0.0175 (5)	0.0020 (4)
C7	0.0237 (5)	0.0276 (6)	0.0247 (6)	−0.0018 (4)	0.0125 (5)	−0.0010 (4)
C8	0.0235 (5)	0.0251 (6)	0.0249 (6)	−0.0002 (4)	0.0142 (5)	−0.0010 (4)
C9	0.0238 (5)	0.0275 (6)	0.0219 (6)	0.0008 (4)	0.0124 (5)	0.0012 (4)
C10	0.0209 (5)	0.0267 (6)	0.0257 (6)	−0.0014 (4)	0.0128 (5)	−0.0035 (4)
C11	0.0274 (6)	0.0254 (6)	0.0299 (6)	−0.0001 (4)	0.0179 (5)	0.0021 (4)
C12	0.0271 (6)	0.0317 (6)	0.0249 (6)	0.0033 (5)	0.0140 (5)	0.0046 (5)
C13	0.0224 (5)	0.0325 (6)	0.0237 (6)	−0.0011 (5)	0.0109 (5)	−0.0003 (5)

### Geometric parameters (Å, º)

**Table:**

O1—C1^i^	1.3992 (12)	C5—H5	0.973 (13)
O1—C1	1.3993 (12)	C6—H6	0.959 (14)
O2—C10	1.3563 (13)	C7—C8	1.4634 (15)
O2—H2A	0.972 (19)	C7—H7	1.012 (14)
N1—C7	1.2755 (14)	C8—C13	1.3935 (15)
N1—C4	1.4250 (13)	C8—C9	1.4028 (15)
C1—C2	1.3831 (16)	C9—C10	1.3848 (16)
C1—C6	1.3849 (15)	C9—H9	0.967 (13)
C2—C3	1.3865 (16)	C10—C11	1.3991 (16)
C2—H2	0.965 (14)	C11—C12	1.3812 (16)
C3—C4	1.3961 (15)	C11—H10	0.988 (14)
C3—H3	0.988 (15)	C12—C13	1.3888 (16)
C4—C5	1.3891 (16)	C12—H11	1.004 (14)
C5—C6	1.3871 (15)	C13—H12	1.007 (13)
			
C1^i^—O1—C1	114.87 (11)	N1—C7—C8	124.35 (10)
C10—O2—H2A	113.5 (11)	N1—C7—H7	120.7 (8)
C7—N1—C4	118.81 (9)	C8—C7—H7	114.9 (8)
C2—C1—C6	120.56 (10)	C13—C8—C9	119.77 (10)
C2—C1—O1	120.77 (9)	C13—C8—C7	117.53 (10)
C6—C1—O1	118.66 (9)	C9—C8—C7	122.65 (10)
C1—C2—C3	120.01 (10)	C10—C9—C8	119.69 (10)
C1—C2—H2	119.8 (9)	C10—C9—H9	117.3 (8)
C3—C2—H2	120.1 (9)	C8—C9—H9	122.9 (8)
C2—C3—C4	120.12 (10)	O2—C10—C9	123.21 (10)
C2—C3—H3	119.8 (8)	O2—C10—C11	116.53 (10)
C4—C3—H3	120.0 (8)	C9—C10—C11	120.26 (10)
C5—C4—C3	119.03 (10)	C12—C11—C10	119.88 (10)
C5—C4—N1	118.41 (9)	C12—C11—H10	121.0 (8)
C3—C4—N1	122.28 (10)	C10—C11—H10	119.1 (8)
C6—C5—C4	120.99 (10)	C11—C12—C13	120.38 (10)
C6—C5—H5	120.6 (8)	C11—C12—H11	118.1 (8)
C4—C5—H5	118.4 (8)	C13—C12—H11	121.5 (8)
C1—C6—C5	119.23 (10)	C12—C13—C8	120.02 (10)
C1—C6—H6	120.2 (8)	C12—C13—H12	120.2 (8)
C5—C6—H6	120.5 (8)	C8—C13—H12	119.8 (8)
			
C1^i^—O1—C1—C2	49.26 (9)	C4—N1—C7—C8	−171.34 (10)
C1^i^—O1—C1—C6	−131.68 (11)	N1—C7—C8—C13	175.25 (11)
C6—C1—C2—C3	1.91 (17)	N1—C7—C8—C9	−2.15 (18)
O1—C1—C2—C3	−179.04 (10)	C13—C8—C9—C10	−1.16 (16)
C1—C2—C3—C4	−0.10 (18)	C7—C8—C9—C10	176.18 (10)
C2—C3—C4—C5	−0.88 (17)	C8—C9—C10—O2	−179.55 (10)
C2—C3—C4—N1	173.02 (10)	C8—C9—C10—C11	0.54 (16)
C7—N1—C4—C5	−145.80 (11)	O2—C10—C11—C12	−179.50 (10)
C7—N1—C4—C3	40.26 (15)	C9—C10—C11—C12	0.42 (17)
C3—C4—C5—C6	0.06 (16)	C10—C11—C12—C13	−0.76 (17)
N1—C4—C5—C6	−174.08 (10)	C11—C12—C13—C8	0.15 (17)
C2—C1—C6—C5	−2.71 (16)	C9—C8—C13—C12	0.82 (17)
O1—C1—C6—C5	178.23 (9)	C7—C8—C13—C12	−176.66 (10)
C4—C5—C6—C1	1.72 (16)		

Symmetry code: (i) −*x*+1, *y*, −*z*+1/2.

### Hydrogen-bond geometry (Å, º)

*Cg*1 is the centroid of the C1–C6 benzene ring.

**Table:**

*D*—H···*A*	*D*—H	H···*A*	*D*···*A*	*D*—H···*A*
O2—H2*A*···N1^ii^	0.972 (19)	1.828 (19)	2.7615 (12)	160.1 (16)
C5—H5···O2^iii^	0.973 (13)	2.431 (14)	3.1121 (14)	126.7 (10)
C12—H11···*Cg*1^iii^	1.004 (14)	2.986 (15)	3.9882 (12)	178.7 (19)

Symmetry codes: (ii) −*x*+1/2, *y*−1/2, −*z*+1/2; (iii) −*x*+1/2, *y*+3/2, −*z*+1/2.
